# Mechanisms of change for interventions aimed at improving the wellbeing, mental health and resilience of children and adolescents affected by war and armed conflict: a systematic review of reviews

**DOI:** 10.1186/s13031-018-0153-1

**Published:** 2018-05-09

**Authors:** Tania Josiane Bosqui, Bassam Marshoud

**Affiliations:** 1 Department of Psychology, American University of Beirut, Riad El-Solh, Beirut, 1107 2020 Lebanon; 2 Danish Red Cross, Mansour, Baghdad, Iraq

**Keywords:** Review, War, Children, Adolescents, Psychosocial, Mechanisms of change

## Abstract

Despite increasing research and clinical interest in delivering psychosocial interventions for children affected by war, little research has been conducted on the underlying mechanisms of change associated with these interventions. This review aimed to identify these processes in order to inform existing interventions and highlight research gaps. A systematic review of reviews was conducted drawing from academic databases (PubMed, PILOTS, Cochrane Library for Systematic Reviews) and field resources (e.g. Médecins Sans Frontières and the Psychosocial Centre of the International Federation of Red Cross and Red Crescent Societies), with extracted data analysed using Thematic Content Analysis. Thirteen reviews of psychosocial or psychological interventions for children and adolescents (< 25 years old) affected by war, armed conflict or political violence were identified, covering over 30 countries worldwide. Qualitative analysis identified 16 mechanisms of change, one of which was an adverse mechanism. Themes included protection from harm, play, community and family capacity building, strengthening relationships with caregivers, improved emotional regulation, therapeutic rapport, trauma processing, and cognitive restructuring; with the adverse mechanism relating to the pathologising of normal reactions. However, only 4 mechanisms were supported by strong empirical evidence, with only moderate or poor quality evidence supporting the other mechanisms. The poor quality of supporting evidence limits what can be inferred from this review’s findings, but serves to highlight clinically informed mechanisms of change for existing and widely used non-specialist interventions in the field, which urgently need rigorous scientific testing to inform their continued practice.

**Electronic supplementary material:**

The online version of this article (10.1186/s13031-018-0153-1) contains supplementary material, which is available to authorized users.

## Background

The detrimental effect on the mental health and wellbeing of children and adolescents exposed to war, armed conflict and political violence has long been established [1], and are increasingly addressed by humanitarian agencies [2, 3]. The higher prevalence rate of psychiatric disorders has been recognized in a number of countries and regions; Posttraumatic Stress Disorder (PTSD) [4], depression, and anxiety [5], disproportionately affect those whose lives are, or have been, affected by war, with particular damaging effects of direct exposure to violence [4]. Beyond psychiatric diagnoses, cognitive, emotional and behavioural concerns have been widely noted, including externalising (outward directed behaviours such as aggression), internalising behaviours (inward directed behaviours such as self-harm) [6] and toxic stress associated with severe and protracted ongoing exposure to extreme violence and instability [7, 8]. Psychosocial and general wellbeing, which the United Nations has defined as children’s ‘health and safety, material security, education, socialization, and their sense of being loved, valued, and included in the families and societies into which they are born’ [9], has also unsurprisingly been shown to be greatly affected by the environment of war [10]. Clinicians and researchers taking a strengths-based approach have additionally helped to understand the often astounding resilience seen in these young people, despite their environment, and the individual, family and community factors that explain these strengths. These include factors such as an internal locus of control, parental support, and community acceptance [11]; all of which can be targeted to build resilience in war-affected populations.

Interventions that aim to mitigate the psychological effects of war on children have been increasingly studied albeit with continuing limitations in scope and quality. Reviews of the effectiveness of these interventions consistently highlight a number of factors that hamper the quality of clinical trials and intervention studies for these populations. Conducting high quality research in current conflicts, often with low or extremely low resources, can prove operationally and ethically difficult, with trials being hampered by outbreaks of violence, population displacement, high drop-out, and security concerns for practitioners travelling to work [12]. Many reviews highlight the number of studies with no control groups [13], lacking culturally and linguistically appropriate standardised outcome measures [14], with poor clarity on theoretical models of interventions [15] or with limited transparency of cultural adaptations of ‘exported’ interventions, developed in one country and delivered in another [13]. Different aims of interventions, be it the treatment of psychiatric disorders such as Trauma-Focused Cognitive Behavioural Therapy (TF-CBT) [16], the promotion of wellbeing such as Psychosocial Structured Activities [17], the prevention of mental health problems and psychiatric disorders such as the school-based intervention Overshadowing the Threat of Terrorism [18], capacity building or resilience building such as the School-Based Psychosocial Program [19], or a mixture of all or parts of these aims, muddies the waters when trying to make conclusive statements about the effectiveness of interventions. This is also in the context of vast diversity in countries, cultures, types of conflict and of war experience, making it conceptually difficult to group interventions together.

The limitations of this research and the difficulty in applying intervention-specific findings in low-resource settings has resulted in a large theory-practice gap [20]. Part of the difficulty in applying research findings in the field is the tendency for intervention research to focus on quantifiable effectiveness for specific manualised interventions, which tends to neglect the wealth of less quantifiable, but valuable, field experience and interventions at different levels, in different cultures and by non-specialist practitioners. Reviews of interventions in the field, both academic and clinical, frequently report on the mechanisms of change employed by the reviewed interventions; the theoretical and operational process by which the targeted outcome is thought to be achieved. For example, many reviews cite the importance of the narration of experience, trauma processing and memory integration and the correction of self-blaming appraisals (e.g. [14]), secure and consistent caregiving (e.g. [13]), and traditional rituals such as for grief or forgiveness (e.g. [10]), as well as highlighting key adverse effects such as the pathologising of normal reactions (e.g. [21]). Reviewing underlying mechanisms of change in these populations, rather than intervention specific effectiveness, could help to connect theory to practice by identifying key therapeutic processes that transcend the diversity of models, approaches and techniques, that could be applied across service settings and socio-political and cultural contexts.

This systematic review of reviews therefore aimed to draw out these key mechanisms of change intrinsic to interventions used across the span of countries, conflict types, and outcome aims. This broad approach accommodates the diversity and overlap of intervention aims and outcomes, as well as the wealth of literature based on fieldwork and clinical experience. Highlighting key therapeutic processes that improve the wellbeing, resilience or mental health of children affected by armed conflict, without a focus on specific intervention efficacy, could be helpful to inform existing interventions on the ground. Furthermore, the inclusion of field-based research and grey literature provides an opportunity to identify mechanisms assumed to work in the field but that require rigorous scientific testing. This is of great importance given the large theory-practice gap for this population and the growing clinical need in the context of unrelenting and horrifying bombardment and displacement of civilian populations in the numerous ongoing wars and conflicts across the world. This review of reviews therefore aimed to use systematic and qualitative methodologies to answer the research question, ‘What are the core mechanisms of change integral to interventions aimed at improving the wellbeing, mental health and resilience of children and adolescents affected by conflict?’

## Method

This review of reviews used systematic methodology drawing on Preferred Reporting Items for Systematic Reviews and Meta-Analyses (PRISMA) guidelines [22] and the Cochrane Handbook for Systematic Reviews (Version 5.1.0) [23]. A review of reviews, rather than of primary studies, was conducted due to the author’s knowledge of numerous existing academic and field reviews of interventions. The protocol was finalised on 8 April 2017 and is available from the authors on request. The review did not seek ethical approval as it drew on secondary sources.

### Search strategy

The databases PILOTS (Published International Literature On Traumatic Stress), PubMed (including Medline), and the Cochrane Library for Systematic Reviews were searched on the 25th April 2017 for reviews of any intervention for populations affected by war, published in English or French, in any country or region, and with no date restrictions. A pilot search using these databases returned all known published reviews and was therefore deemed sufficient. The title search terms were: [child OR adolescent] AND [war OR armed conflict OR community violence OR political violence] AND [intervention OR treatment OR therap*] AND [psychological OR psychosocial OR mental health]. The search results were filtered by ‘review’ in PubMed and PILOTS. Due to limited time and resources, the PubMed search was restricted to a search of titles, whilst PILOTS and Cochrane remained as a search of titles, abstracts and keywords. Grey literature was also searched manually on the 27th April 2017, to reduce publication bias and include field expertise, using the online library archives and/or publication lists of the War Trauma Foundation, War Child, Médecins Sans Frontières (MSF), the Psychosocial Centre of the International Federation of Red Cross and Red Crescent Societies (IFRC), Save the Children, and Médecins du Monde, with the search term [psychosocial] OR [mental health].

### Inclusion criteria

This review of reviews included children, adolescents and young people aged 25 or younger, who have experienced or continue to experience war, armed conflict or political violence, and any psychological or psychosocial intervention that aims to improve the wellbeing, mental health and/or resilience of children and adolescents, in any format, and at every level of the IASC (Inter-Agency Standing Committee) 4-tiered pyramid model for mental health and psychosocial support in emergencies [24]. For the purposes of this review, intervention aims were categorised as prevention, promotion, and/or treatment. Prevention refers to interventions that aim to prevent mental ill health or distress, such as universal psychoeducation. Promotion refers to interventions that aim to promote wellbeing, resilience and optimal development, such as targeted school-based interventions. Treatment refers to interventions that aim to treat mental ill health and psychiatric disorders, such as Trauma-Focused Cognitive Behavioural Therapy (TF-CBT) [25] and Narrative Exposure Therapy for children (KidNET) [26, 27]. Some interventions may also have mixed aims, particularly given the interaction between the prevention of distress and promotion of wellbeing.

Children affected by single terrorism-related events in non-war affected countries were excluded, as well as displaced populations in non-war affected countries. Displaced populations that continued to live in their, or another, country affected by war were included. Reviews that had a wider scope but provided sub group analysis of children or adolescents affected by war were also included.

### Data screening and extraction

Records were first screened by title by the first author to exclude obviously irrelevant studies, abstracts and full texts were then independently duplicate screened by the first and second authors. An interrater agreement rate of 96.08% was reached, using Cohen’s kappa statistic (k = 0.89, SE = 0.14). Discrepancies were resolved through discussion and consensus.

Included reviews were screened by the first author and all sentences or paragraphs that referred to mechanisms of change relating to any of the included interventions were extracted, along with data on the number and ages of participants, the countries and settings, the types of conflict, the types of interventions, outcome measures, and the type and quality of evidence supporting the mechanisms. Sentences or paragraphs that referred to impediments to change, such as intervention adverse effects, were also extracted. Mechanisms were cited in reviews as part of a synthesis of findings drawn from their included studies.

‘Mechanisms of change’ were defined as the process or steps responsible for a therapeutic outcome. Mechanisms explain *how* change occurred [28]. For the purposes of this study, the term was used interchangeably with mediators of change, which refers to variables that account for the relationship between an intervention and a therapeutic outcome. In the included reviews, mechanisms were referred to as therapeutic processes, contextual processes (natural interventions), mechanisms of change, mechanisms of counselling, treatment processes, factors that produce change, or mediators of change. This definition therefore excludes moderators of change (characteristics that influence the magnitude of the relationship, such as age or gender) and protective factors, as well as practice elements, clinical techniques, strategies, or intervention aims.

### Data analysis

Due to insufficient quantitative testing of mechanisms in the literature [28], a qualitative data analysis was conducted. Data was analysed within Malterud’s framework for qualitative research [29] using Newell and Burnard’s Thematic Content Analysis [30]. Thematic Content Analysis is rooted in Grounded Theory and has been used extensively in public health research [31]. Briefly, extracted texts were read and re-read firstly with open coding for emerging themes, in order to build an initial coding framework. Secondly, the coded text was re-read with duplicates removed and overlapping codes amalgamated into core themes. Core themes were then organised into a pre-determined order using the 4 tiers of the IASC pyramid [24]: 1) basic services and security, 2) strengthening family and community support, 3) focused non-specialist support, and 4) specialist support. Duplicate analysis of the extracted data was conducted by the second author using the same protocol, to maximise the validity and reliability of the results. A reflection diary was used to support researcher reflexivity and consideration of potential researcher bias.

To further explore the scope and quality of research, two additional analyses were conducted. The first to identify the global coverage of the identified research, using a map derived from the Global Peace Index, from 2007 when data was first available, until the most recent publication in 2016. The Index categorises countries based on 23 indicators of internal violent conflict, international war, political insecurity and militarisation [32]. The map was amended to display countries affected by conflict (a score of 2.38 or more (low or very low peace) in any year of the index period), and countries in which at least 1 study has been conducted on psychological interventions for children, according to the results of this review of reviews. Some reviews included studies on refugee populations in high-income countries (e.g. Kosovan and Roma refugees in Germany, [14]) or single terrorist attacks (e.g. 9/11 in the United States, [6]), and these countries were excluded from the map. Copyright for use of the original map was granted by the Institute for Economics and Peace. Secondly, in order to support and inform the qualitative interpretations of data, citations for each identified mechanisms of change were counted and organised into prevention, promotion and treatment intervention categories, stratified by mechanism quality of supporting evidence ratings.

### Quality assessment and risk of bias

The quality of each included review was assessed using the AMSTAR Checklist (A Measurement Tool to Assess Systematic Reviews, Copyright © 2015 AMSTAR All Rights Reserved, [33]). This tool assesses reviews based on quality criteria, including duplicate study selection and publication bias, and provides a quality rating (low, moderate and high).

The quality of supporting evidence for the mechanisms of change was also assessed. Extracted data on the type of supporting evidence was compiled for each mechanism. A rating scale was developed based on the recommendations of Kazdin [28]. The quality of evidence was rated as low if the mechanism was described based on case studies, qualitative research, clinical experience, cross-sectional research, or program evaluations. A moderate quality rating was assigned if the mechanism was supported by quantitative data from intervention controlled trials. A high quality rating was given when the mechanism was supported by quantitative data specifically testing the mechanism, such as through mediational analysis.

## Results

The database and grey literature search produced a total of 2359 records from which 13 reviews were included (for details of the review process see Additional file 1: Figure S1). Details of the included reviews are displayed in Table 1 (for excluded studies see Additional file 2: Table S1). Within the included reviews, 7 were systematic reviews of quantitative studies, 3 were systematic reviews of quantitative and qualitative studies and 3 were unsystematic narrative reviews. The reviews included studies up until 2017 and covered a diversity of countries, age groups and war settings, with pooled sample sizes ranging from 730 to 32,046.

**Table 1:** Details of included reviews

Review authors and year	Review design	Years of inclusion	Number of studies included	Total number of participants	Age of participants	Countries and regions included	Type of conflict included	
Ager et al. [17]	Systematic review of quantitative studies	1997–2012	10	NR	Age 4 to late teens	Darfur, Indonesia, Myanmar, Palestine, Serbia, Sudan (north), Uganda, Yemen	Humanitarian and emergency contexts including conflict and natural disasters	
Apfel & Simon [1]	Unsystematic narrative review	NR	NR	NR	Children	Argentina, Basque, Bosnia, Cambodia, Ethiopia, Israel, Iraq, Israel, Lebanon, Mozambique, Palestine, Vietnam, worldwide holocaust survivors	War and armed conflict	
Betancourt et al. [14]	Systematic review of quantitative studies	1990–2011	53	32,046	Children, Adolescents & Youth	Angola, Bosnia, Burundi, Croatia, El Salvador, Indonesia, Israel, Kosovan & Roma refugees in Germany, Kosovo, Lebanon, Nepal, Palestine Rwandan & Somali refugees in Uganda, Serbia, Sierra Leone, Sri Lanka, Sudan, Sudanese & Sierra Leonean refugees in USA	Post-conflict settings or a setting with protracted political violence	
Brown et al. [37]	Systematic review of quantitative studies (RCTs or CTs)	1840–2015	28	5457	≤ 24	Bosnia, Burundi, Democratic Republic of the Congo, Indonesia, Iran, Kosovo, Lebanon, Nepal, Palestine, Rwanda, Sierra Leone, Sri Lanka, Uganda	Areas affected by recent or ongoing conflict (post-World War II), including former child soldiers, and in a LMIC	
Gillies et al. [48]	Systematic review of quantitative studies (RCTs or quasi-RCTs)	1974–2015	13	2936	≤ 18	Bosnia, Burundi, Democratic Republic of the Congo, Indonesia, Israel, Palestine, Sierra Leone, Sri Lanka	Sub-group analysis based on type of trauma: Community violence or war	
Jordans, Pigott & Tol [36]	Systematic review of quantitative and qualitative studies	2009–2015	24	4848	Children & Adolescents	Bosnia & Herzegovina, Burundi, Democratic Republic of the Congo, Indonesia, Palestine, South Sudan, Sri Lanka, Sudan, Uganda	War, armed conflict or community violence, and a LMIC	
Jordans et al. [13]	Systematic review of qualitative and quantitative studies	1991–2008	66	1824	Child & Adolescents	Afghanistan, Angola, Azarbaijan, Chechnya, Croatia, Bosnia, Ethiopia, Guatemala, Iraq, Kosovo, Mexico, Mozambique, Rwanda, Sierra Leone, Sri Lanka, Uganda, West-Bank & Gaza, Zimbabwe	War, armed conflict or community violence, and a LMIC	
Kalskma-Van Lith [49]	Unsystematic narrative review	NR	NR	NR	Children & Adolescents	NR	War-affected areas	
O’Sullivan, Bosqui & Shannon [15]	Systematic review of quantitative studies (RCTs or CTs)	1806–2014	17	4956	Youths 5–25	Bosnia, Burundi, Democratic Republic of the Congo, Indonesia, Israel, Lebanon, Nepal, Palestine, Sri Lanka, Uganda	Protracted armed conflict or political violence	
Peltonen & Punamäki [6]	Systematic literature review of quantitative studies (RCTs, quasi-experimental or experimental)	1980–2008	19	1349	< 18	Bosnia, Croatia, Gaza, Kosovan refugees in Germany, New York, Refugees from Croatia, Bosnia and Herzegovina in Slovenia Refugees in London, Somali refugees in Boston, Somali refugees in Uganda, Sri Lanka	Armed conflict, war, military violence, terrorism or living as refugees	
Tol, Song & Jordans [11]	Systematic review of quantitative and qualitative studies	Up until 2012	53	730	< 18	Croatia, Afghanistan, Gaza, Sierra Leone	Armed conflict, war or political violence, and in a LMIC	
Tol et al. [20]	Systematic review of quantitative studies (RCTs)	Treatment, prevention and promotion	Up until Sept 2010	19	4239	Sub-group analysis: Children & adolescents	Armenia, Bosnia & Herzegovina, Burundi, Ethiopia, India, Indonesia, Iran, Lebanon, Nepal, occupied Palestinian territories, Rwanda, Sri Lanka, Uganda	Humanitarian disaster, war, armed conflict or political violence, in a LMIC
War Child [21]	Unsystematic narrative review	Treatment, prevention and promotion	NR	NR	NR	Children & adolescents	NR	War-affected areas

*CTs* Controlled Trials, *LMIC* Low or Middle Income Country, *NR* Not reported, *RCTs* Randomised Controlled Trials

**Fig. 1:**
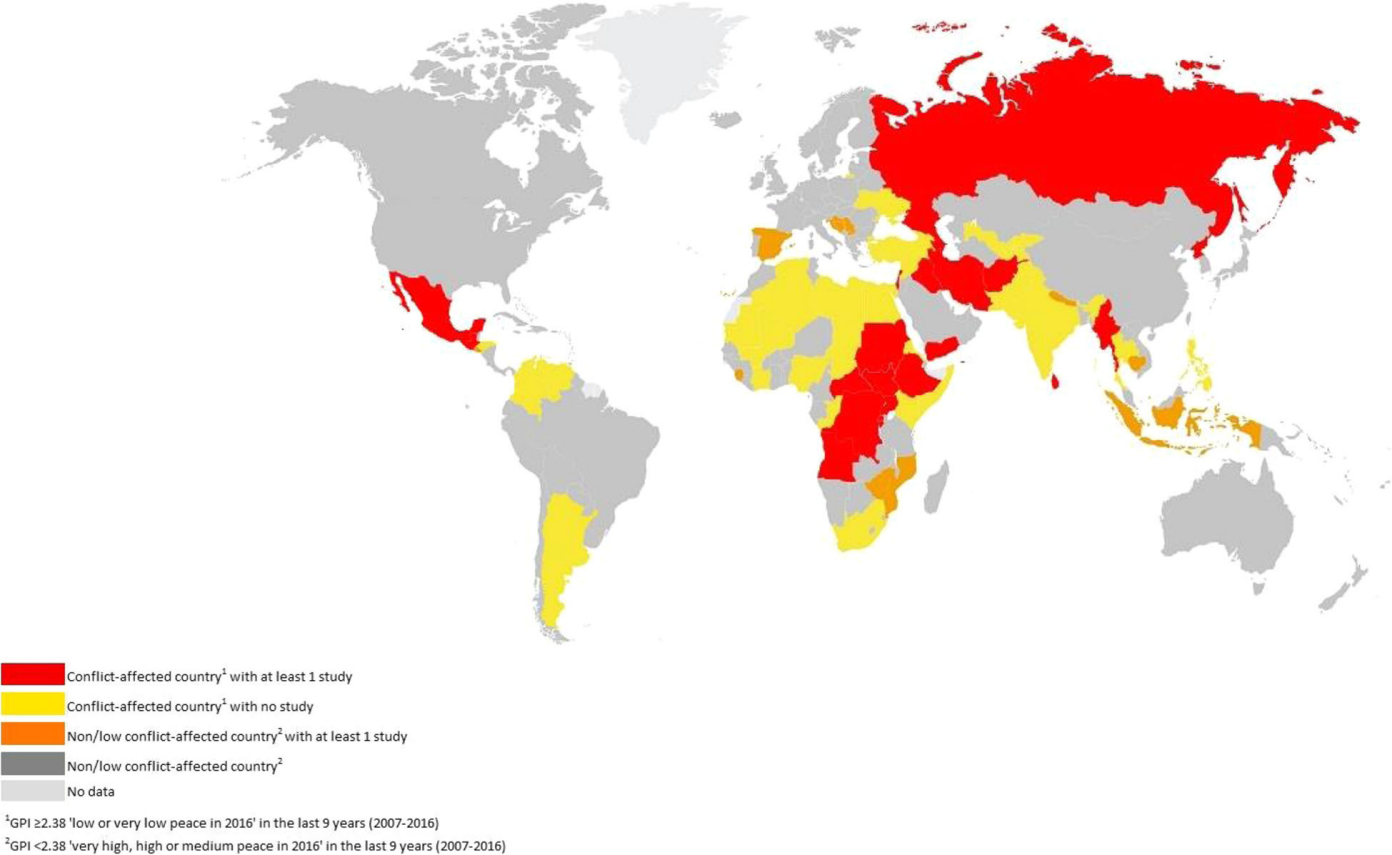
Map displaying countries affected by conflict using the Global Peace Index (GPI, 2007–2016), highlighting countries that have and have not had research conducted on psychosocial interventions for children

The global coverage of the reviews is displayed in Fig. 1 and indicates that there are a number of countries and regions affected by conflict that have yet to have a study conducted on psychological interventions for children, predominantly in North Africa, south Asia and South America. Countries with brutal violent conflicts such as the Central African Republic, Syria and Somalia were not included in a single review. Some reviews covered countries not identified as conflict-affected within the last 9 years (according to the Global Peace Index 2007–2016), although this was largely due to sub-regional political violence such as in Poso, Indonesia ([34], reviewed in 15) or follow ups of historical conflicts such as the Spanish Civil War ([35], reviewed in 1).

The AMSTAR quality assessment results are displayed in Table 2. The assessment shows that 5 reviews were of low quality, 6 of moderate quality and 2 of high quality. Studies were most commonly marked down for not providing protocols, not providing a list of excluded studies, and not assessing publication bias.

**Table 2:**
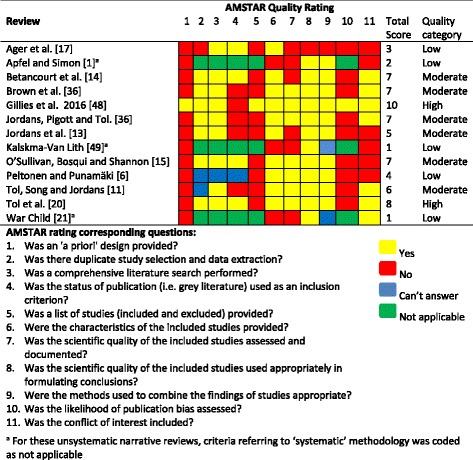
Quality assessment of reviews using AMSTAR [33]

### Mechanisms of change

Out of the 13 reviews, only 6 referred to the mechanisms of change of their included interventions. Seven reviews were therefore not included in the analysis. In total, 16 core mechanisms (including 1 adverse mechanism) spanning the 4 tiers of the IASC model and different intervention aims were identified, and are outlined in Table 3. A quality assessment of these mechanisms’ supporting evidence rated 7 mechanisms as low, 5 as moderate, and 4 as high quality. Only one review [36] referred to two studies that statistically tested the mechanisms of change.

**Table 3:** Outline of mechanisms of change for interventions aimed at improving the wellbeing, mental health and resilience of children and adolescents affected by war and armed conflict

Mechanisms		Cited reviews	Outcomes	Evidence Quality
Basic services and security				
1	Creating safety and protection from harm	Ager et al. [17]	Protection outcomes (sense of safety, exual exploitation and rape, physical injuries, referrals, reporting); social and emotional wellbeing	Low: Program evaluations
2	Playing	Apfel and Simon [1]; Betancourt et al. [14]	Resilience; wellbeing; self-confidence; emotional regulation	Low: Case or cross-sectional studies
Strengthening family and community support				
3	Community capacity building	Apfel and Simon [1]; Ager et al. [17]; Peltonen & Punamäki [6]	Knowledge of protection systems; sense of order and sanity; PTSD; improved psychosocial wellbeing	Low: Program evaluations
4	Increasing social support	Apfel and Simon [1]; Peltonen & Punamäki [6]	Resilience; PTSD; improved psychosocial wellbeing	Low: Program evaluation and clinical experience
5	Family and caregiver capacity building	Apfel and Simon [1]; Jordans, Pigott and Tol [36]	Ability of caregivers to provide consistent and reliable care; depression; PTSD; anxiety symptoms; hope	High: Statistical testing of mechanism
6	Family and caregiver relationship strengthening.	Apfel and Simon [1]; Betancourt et al. [14]; Jordans, Pigott and Tol [36]	Further traumatic experience; psychosocial functioning; mental health; maternal mental health; depression; PTSD; anxiety symptoms; hope	High: Statistical testing of mechanism
7	Engaging with values, traditions, religious and non-religious beliefs, and ideologies	Apfel and Simon [1]; Betancourt et al. [14]; Tol, Song and Jordans [11]	Morale and healing; maintaining the right to be alive despite suicidal despair; drive to survive; community and personal restitution; empowerment; reintegration into communities; wellbeing	Low: Qualitative studies or clinical experience
Focused non-specialist support				
8	Learning about the presenting problem, medication, and how to access services (psychoeducation)	Betancourt et al. [14]	Medication compliance; access to services; distress	Moderate: Statistic testing but of intervention not mechanism
9	Learning stress management skills	Peltonen & Punamäki [6]	PTSD; psychosocial wellbeing	Moderate: Statistic testing but of intervention not mechanism
10	Emotional regulation and bearing negative emotions	Apfel and Simon [1]; Peltonen & Punamäki [6]	Chances of survival; resilience	Low: Program evaluation and clinical experience
11	Problem solving	Jordans, Pigott and Tol [36]	Depression; PTSD; anxiety symptoms; hope	High: Statistical testing of mechanism
12	Learned helpfulness	Apfel and Simon [1]; Betancourt et al. [14]	Helplessness; wellbeing	Moderate: Statistical testing but of intervention not mechanism
Specialist support				
13	Adverse mechanism: Pathologising normal reactions	Apfel and Simon [1]	Alienating participants	Low: Clinical experience
14	Trauma processing through narratives, exposure, dreaming or play	Apfel and Simon [1]; Betancourt et al. [14]; Jordans, Pigott and Tol [36]; Peltonen & Punamäki [6]	Memory integration; PTSD; depression; PTSD; anxiety symptoms; hope; psychosocial wellbeing	Moderate: Statistical testing but of intervention not mechanism
15	Restructuring unhelpful cognitions and appraisals	Peltonen & Punamäki [6]	PTSD; psychosocial wellbeing	Moderate: Statistical testing but of intervention not mechanism
16	Therapeutic rapport	Jordans, Pigott and Tol. [36]	PTSD; anxiety symptoms; hope	High: Statistical testing of mechanism

#### Creating safety and protection from harm

One review [17] cited the need to first and foremost protect children from harm, to create a protective environment and a sense of safety, in order to prevent further traumatisation, exploitation, and to promote wellbeing and mental health. Ager et al. [17] describe how ‘the building and strengthening of a protective environment for children vulnerable to abuse, exploitation and/or violence is paramount to effective [intervention].’ The evidence supporting this mechanism is low, however, as it is based primarily on program evaluations.

#### Playing

Two reviews [1, 14] cited the need for children to play in order to create a normal environment, to safely act out and explore traumatic memories and their meanings, to build relationships, using drama, music, role-playing and drawing, and to counterbalance stressful experiences. Apfel and Simon [1] describe how ‘interventions and programs that encourage and allow children to play, including playing out some of the traumatic events to which they have been subjected, may have a considerable impact on the child's ability to cope.’ The evidence supporting this mechanism is low, however, as it is based primarily on case or cross-sectional studies.

#### Community capacity building

Three reviews [6, 14, 17] cited community capacity building and the strengthening of community protective mechanisms for children as an important mechanism of change, through Child Friendly Spaces, greater community contact and reporting of violations of safety, an improved sense of community efficacy, stronger school systems and social networks, and good community cohesion. Apfel and Simon [1] describe ‘the tremendous importance of school in establishing and re-establishing some order and sanity in the lives of children traumatized by violence. School can provide the stabilizing framework in which the child's imaginative and cognitive skills can safely grow, or grow in relative safety.’ The evidence supporting this mechanism is low, however, as it is based primarily on program evaluations.

#### Increasing social support

Two reviews [1, 6] cited the importance of increasing social support, sourcing social support outside of immediate family, whose capacity may be stretched, and improving social skills to boost self-esteem, interpersonal deficits and access to social supports. ‘Resilient children have a knack,’ writes Apfel and Simon [1], ‘for turning to adults other than parents for guidance and resources if they cannot find such support in their own families.’ The evidence supporting this mechanism is again low, however, as it is based primarily on program evaluations and clinical experience.

#### Family and caregiver capacity building

Two reviews [1, 36] cited the need to support families, caregivers and practitioners in order to improve their ability to support children, through psychoeducation, dialogue, and through self-care. Caregivers are affected by the same war and violence affecting the children they care for, with the addition of containing the distress of children and their own childhood traumatic experiences. Statistical testing in two studies cited by Jordans, Pigott and Tol [36] provides high quality evidence for this mechanism.

#### Family and caregiver relationship strengthening

Three reviews [1, 14, 36] cited the strengthening of family and therapeutic relationships, of involvement in interventions, and of improved consistency of caregiving, particularly during periods of active conflict, for the long term wellbeing of children. This was also cited within the context of looking beyond traumatisation to the daily experience of children within the context of collectivist cultures, where family relationships are a core resource. Statistical testing in two studies cited by Jordans, Pigott and Tol [36] provides high quality evidence for this mechanism.

#### Engaging with values, traditions, religious and non-religious beliefs, and ideologies

Three reviews [1, 11, 14] cited the engagement in traditional, religious and political belief systems as important to enhance child wellbeing, by building hope and strength, connecting to culture, and restoring a sense of safety and normalcy. Also cited was the role of values like hope, strength, perseverance, forgiveness, honour and trust, and of culturally specific values such as *Sumud* in Palestine (meaning connection to the land, steadfastness, and the struggle to persist), *Kwizerana* in Rwanda (meaning family trust), *Tarbia* in Afghanistan (meaning a strong sense of morality), as well as customs such as cleansing rituals in Angola for the reintegration of former child soldiers into communities. Betancourt et al. [14] describe how ‘in many settings, traditional healing practices make critical contributions to social healing in the context of war. For instance, in Zimbabwe, Zezuru healers are known to engage family and community members in groups, draw out concerns over children's problems, facilitate reconciliation in and between families, and create a restorative climate.’ The evidence supporting this mechanism is low, however, as it is based primarily on qualitative studies or clinical experience.

#### Learning about the presenting problem, medication, and how to access services (psychoeducation)

One review [14] cited the mechanism of learning about symptoms of mental ill health and improving awareness about how to access services as a mechanism to improve mental health, especially when combined with skills building and counsellor contact. Betancourt [14] describes how ‘classroom-based programs that combine psychoeducation, skills building, and supportive counselor contact may be adequate to reduce distress in war-exposed youths living in low-resource settings.’ The evidence for this mechanism is moderate as it is based on controlled trials of interventions, but without specific testing of the proposed mechanism.

#### Learning stress management skills

One review [6] cited improving stress management skills as a key mechanism in promoting wellbeing, and preventing and treating mental ill health, including ‘relaxation techniques, good sleep habits… building safe settings, [and] setting positive goals,’ which function by reducing distressing symptomology of PTSD, as well as enhancing effective coping, increasing body and emotional self-awareness, and improving sleep. The evidence for this mechanism is also moderate as it is based on controlled trials of interventions, but without specific testing of the proposed mechanism.

#### Emotional regulation and bearing negative emotions

Two reviews [1, 6] cited the mechanism of improving emotional regulation, and the reduction of avoidance of negative or uncomfortable emotions, in order to promote wellbeing and prevent and treat mental ill health; by recognising, tolerating and responding to emotions as opposed to the natural tendency for temporary relief through avoidance, denial or suppression; as well as safely re-processing painful, shameful and overwhelming feelings. Cultural differences in the acceptability of expressing emotions were also cited however, as well as the adaptive mechanism of avoidance during ongoing emergencies, in order to concentrate on survival and defer emotional processing to a safer time. Apfel and Simon [1] describe the benefits of emotional flexibility, where there ‘is some ability to defer or defend against some overwhelming anxiety or depression when emergency resources are needed. This may mean compartmentalizing the pain and deferring the experience of overwhelming emotion until a time or situation when it is safer to experience it.’ The evidence supporting this mechanism is low, however, as it is based primarily on program evaluations and clinical experience.

#### Problem solving

One review [36] identified the process of ‘active problem solving’ as a positive mechanism for children’s mental health and psychosocial wellbeing as part of focused non-specialist interventions. The evidence for this mechanism is high as it has been statistically tested through mediational analysis.

#### Learned helpfulness

Two reviews [1, 14] cited the process of altruism and helping others as a vehicle to promote wellbeing and prevent mental ill health, through an improved sense of purpose and increased internal locus of control, such as a preventative intervention for young children which encourages being a responsible caregiver for a toy dog. This mechanism is described by Apfel and Simon [1] as ‘learned helpfulness’ in contrast to the well-known phenomenon of ‘learned helplessness.’ They describe how altruistic acts create ‘a sense that "you may be helpless right now to stop a bomb from falling, but you are not helpless to deal with its human consequences"’ and support it with studies from Beirut, that found that ‘children instructed to use the interval between shellings to go out and bring food to an invalid relative, instead of using the time to watch television, did much better.’ The evidence for this mechanism is of moderate quality as it is based on controlled trials of interventions, but without specific testing of the proposed mechanism.

#### Adverse mechanism: Pathologising normal reactions

In the context of providing specialist support, one review [1] cited an adverse mechanism in which children’s wellbeing and mental health can be harmed by pathologising normal and adaptive responses to the extreme stresses of war environments. Apfel and Simon [1] state that ‘interventions specifically labelled as "psychological’, let alone "psychiatric," can alienate most of the people they are intended to help…survivors of terrible traumas such as the Holocaust or the Cambodian genocide have conveyed that they have already been labelled, categorized, and declared deviant, if not sub-human. These groups do not need any further psychiatric categorizing.’ The authors strongly recommend avoiding this by blending interventions into wider welfare programmes: ‘Combining psychosocial interventions with basic health and welfare interventions, therefore, tells both the clients and the providers that to be upset is expectable and that such responses are not deviant.’ The evidence for the adverse effect is of low quality however, as it is based on descriptions of clinical experience.

#### Trauma processing through narratives, exposure, dreaming or play

Four reviews [1, 6, 14, 36] described some form of trauma processing as a mechanism to treat traumatic stress, through narration or prolonged exposure to help to re-organize and integrate traumatic autobiographical memories. The technique to deliver this mechanism of change differs across interventions; through storytelling in KidNET in which children tell their whole life story, with detailed exploration of all traumatic memories; through imaginal exposure or in vivo exposure in CBT in which children retell specific traumatic events or face reminders of these events; through play re-enactment; or through dream work and guided imagery. Peltonen & Punamäki [6] describe the latter as techniques that enable a ‘rich, structurally coherent and healing symbolic process.’ The evidence for this mechanism is of moderate quality as it is based on controlled trials of interventions, but without specific testing of the proposed mechanism.

#### Restructuring unhelpful cognitions and appraisals

One review [6] describe the mechanism of altering and restructuring unhelpful, upsetting and unrealistic thoughts, interpretations and appraisals to treat and prevent traumatic stress, by both the correction of biased interpretations and the reframing of causal attributions (e.g. self-blame). Peltonen & Punamäki [6] describe how this process helps in ‘making sense of trauma…empowering coping skills and integrating of fragmented and intrusive thoughts and feelings into a more coherent experience.’ The evidence for this mechanism is again of moderate quality as it is based on controlled trials of interventions, but without specific testing of the proposed mechanism.

#### Therapeutic rapport

One review [36] cited the role of therapeutic rapport in treating mental ill health, specifically the development of a trusting therapeutic relationship and a safe environment for disclosure of traumatic experiences. In contrast, an adverse role was cited for therapeutic relationships which take on a moralistic stance. Jordans, Pigott and Tol [36] describe positive correlations in a mediation analysis for ‘counsellor demonstration of reflective involvement, the opportunity to express emotion, and the absence of moralistic behaviour.’ The evidence for this mechanism is rated as high as it is based on mediational analysis of the mechanism.

**Fig. 2:**
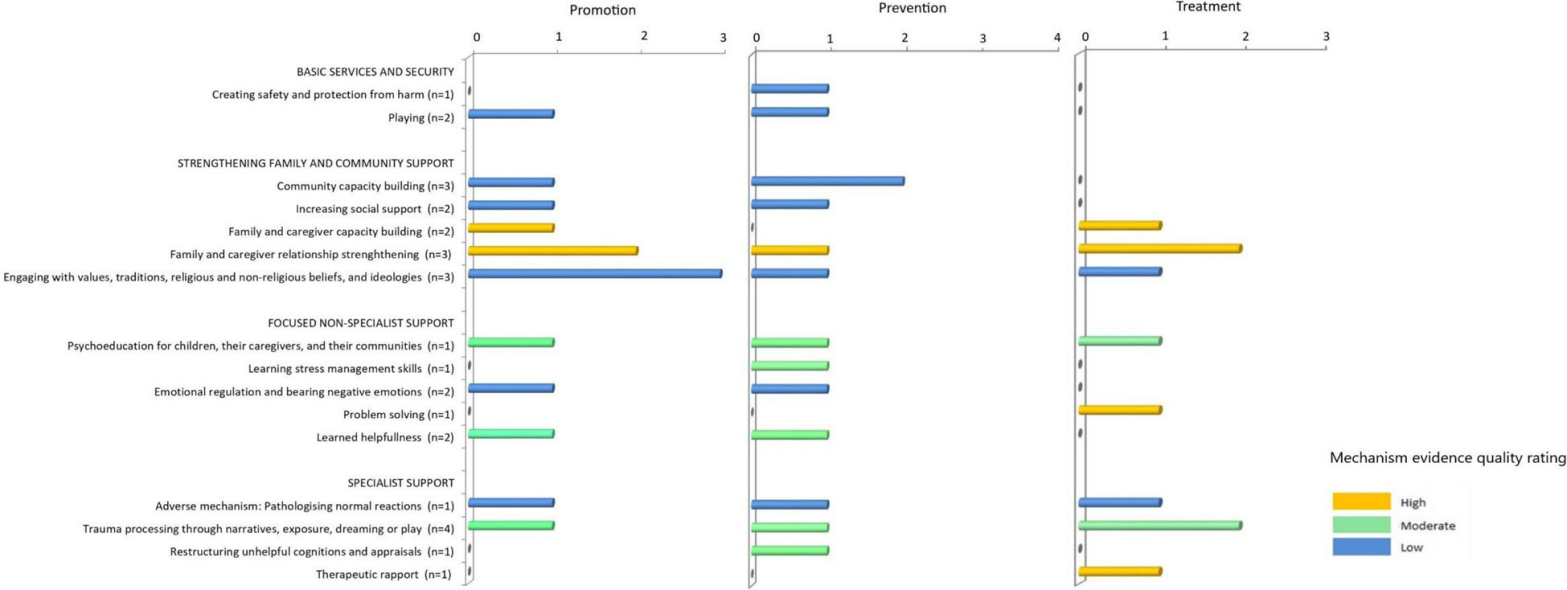
Frequency of mechanisms of change citations displayed separately for promotion, prevention and treatment interventions, colour coded by quality of evidence

These mechanisms with their respective quality ratings are displayed in Fig. 2. Overall, the quality of evidence is poor, with few studies testing mechanisms statistically. High quality evidence was found only for family capacity building, relationship strengthening, problem solving, and therapeutic rapport. Mechanisms at lower levels of the IASC pyramid (basic services and security, and strengthening family and community support) such as protection from harm, play, and capacity building had the poorest quality of evidence. Trauma processing was the most cited mechanism, and was included at least once for each intervention type.

### Subjective reflection and researcher bias

Researcher reflective notes showed three core considerations. Firstly, the utility of interventions (and intervention research) aiming to treat children for post-traumatic stress disorders was raised because of the reliance on *post* trauma literature and its application to populations experiencing ongoing and indefinite terror. Secondly, the eighth mechanism of engaging with traditions was worded carefully due to concern raised about cultural biases and norms around traditional healing, particularly as most reviews were conducted by authors based in European or North American countries. Finally, the difficulty in differentiating mechanisms from techniques and protective factors was noted. Reviews often highlighted this lacking detail and clarity on underlying processes in their included studies.

## Discussion

This review of review aimed to identify the key mechanisms of change intrinsic to psychosocial and psychological interventions for the wellbeing, mental health and resilience of children and adolescents affected by armed conflict in order to help inform existing interventions and highlight research gaps. The mechanisms of change highlighted by this review indicate processes for prevention, promotion and treatment at every level of the IASC [24] model, drawing on individual, family and community resources. The review found a conceptual role of safety and play; of community and family capacity building; and of focused support including stress management skills, problem solving, emotional regulation, and altruism. For interventions at the specialised level, designed to treat mental ill health and psychiatric disorders, the findings of this review highlight the role of trauma processing (through narrative storytelling or exposure), cognitive restructuring and therapeutic rapport, as well as the risk of harm through the pathologising of normal reactions. However, the results of this review of reviews also found predominantly poor quality of supporting evidence, particularly for interventions at the basic services and security level. Gaps in the testing of intervention mechanisms were of particular concern, with only one review citing mediational analyses, as well as a neglect of specific countries, regions and contexts affected by war and conflict that limits the generalisability of research findings to these settings.

The basic psychosocial needs of children in the midst of volatility, insecurity and violence to be kept safe from harm, to maintain some form of normality and routine, and to engage in recreational and playful activities was clearly described in the included reviews, particularly from lower quality reviews based on program evaluations, and conducted by or in collaboration with non-governmental actors in the field. This is evidence of a characteristic of the research-practice gap highlighted by Tol et al. [20] in which the most commonly used interventions in the field are the least well studied, and supports the need for better quality research on these most basic and necessary of interventions. This is even more important in the context of global mental health and LMIC, where interventions need to be both efficacious and readily available for large-scale implementation in low resource and insecure settings [37, 38]. Mechanisms associated with the strengthening of family and community support had some good supportive evidence, particularly for improving caregiver capacities to provide for the needs of children, and to strengthen family relationships, both of which were supported by mediational analysis. They highlight the many ways in which non-individual community wide public mental health interventions can be helpful in promoting wellbeing, preventing, and treating mental ill health in children. Promoting positive change through strengthening children’s relationships with family and caregivers was highlighted as particularly important during active conflict, which is in keeping with research on the protective effect of attachment security and parental support during ongoing conflicts [39, 40]. The promotion of wellbeing through the support of existing traditional or religious resources was widely cited, describing not just the importance of locally derived existing community based interventions (e.g. [19]), but also of helpful concepts that can be incorporated into interventions to promote resilience in a meaningful way, such as Tol et al. ([11], p449) example of the role of ‘Sumud’ for the wellbeing of children in the occupied Palestinian territories, meaning ‘the struggle to persist.’ Despite being widely cited, the evidence for this mechanism is poor, relying on qualitative research or clinical experience, and requires further research and direct testing.

Mechanisms as part of more focused support identified the underlying rational for frequently used techniques such as psychoeducation, stress management, and problem solving, to improve self-understanding, awareness of common psychological reactions to extreme stress, and positive coping in children affected by war and mass violence, although only problem solving was supported by high quality evidence. The review also identified less studied mechanisms, with only low or moderate supporting evidence, including the bearing of emotional distress, and altruism to improve an internal local of control and sense of purpose. The bearing of emotion is a complex mechanism; where adaptive responses may be counter-intuitive. The natural tendency to avoid difficult, shameful or painful emotions may not be detrimental to children’s psychological wellness during or in the immediate aftermath of a disaster as it enables children to concentrate on survival and escape [1]; and it may also encourage concrete thinking during exposure which is associated with a lower risk of developing PTSD in the longer term [41]. However, reviews described the need to face and bear these emotions *appropriately* at a safe time, in order to assist in the emotional processing of events and their meaning. The mechanism warrants further research with adequate statistical testing, as it currently rests on poor quality evidence. The unusual mechanism of altruistic acts, or ‘learned helpfulness’, with moderate supporting evidence, is reported to promote a sense of purpose and control, both of which have been associated with better outcomes for children affected by war [20, 42].

The most cited mechanism for focused interventions for the treatment of PTSD was the processing of trauma through narrative or exposure techniques. The need to identify, order, attend to and integrate intrusive traumatic memories is a well-established mechanism in non-conflict settings (e.g. in the cognitive model of PTSD by Ehlers & Clarke [43]) and this review found moderate evidence to support its continued practice in war settings. The mechanism proposes that war experiences that are overwhelming and terrifying in nature, particularly if they are fragmented (for example, due to losing consciousness or high anxiety), are not processed into autobiographical memory, remaining unprocessed and experienced as a present and ongoing threat. This manifests as intrusive images and flashbacks, nightmares and re-experiencing. Attempts to suppress these intrusions only serve to increase their frequency [43]. Traumatic processing therefore involves breaking down and ordering traumatic memories, paying attention to them, through playing, telling stories, recounting the memory, drawing, and working through nightmares, in order to integrate memories into the past. Many interventions (e.g KidNET, TF-CBT) also involve the checking and re-checking of recounted experiences, to ensure their accuracy, improve integration and to highlight and amend unrealistic or unhelpful interpretations of the events. The latter was identified as a separate core mechanism for the prevention of PTSD and other disorders, termed cognitive restructuring. Much of the literature that supports the trauma processing mechanism is derived from countries in which traumatic experiences are one-off (a car accident) or in the past (childhood sexual abuse) with a strong assumption of ‘post-trauma’ in which the individual is now safe, either removed from harm (away from an abuser) or within normal range (getting back into a car) [43]. The usefulness of this assumption in war settings is clearly limited, with a sense of ongoing threat very much based in reality [8].

The mechanism with the best evidence within specialist support was therapeutic rapport, operationalised as reflective practice, a safe environment to express emotion, and no moralistic or judgemental behaviour. This finding is in keeping with past research in non-war settings, which has found therapeutic rapport to be the strongest mechanism of therapeutic change, followed by specific treatment and client factors [44, 45]. However, past research has not replicated this finding in child and adolescent populations due to insufficient mediational analyses [46]. The findings of this review, however, do indicate that therapeutic rapport remains an important mechanism of change for children and adolescents, at least in the context of armed conflict.

This review identified one adverse mechanism, albeit based purely on clinical experience; pathologising children who are experiencing normal reactions to terror. The risk of increasing post-traumatic reactions by implementing trauma processing too early after an incident has long been established [47] and has since been avoided by humanitarian agencies who opt instead for wider public mental health interventions like Psychological First Aid [48]. However, within the context of multiple, sustained and prolonged exposure to mass violence, the merits of trauma processing remains unclear. Some interventions that promote wellbeing and prevent PTSD aim to reduce longstanding post traumatic symptoms even in the context of ongoing threat, treating past trauma without an assumption of current safety (e.g. [34]). Such interventions merit further research, and are of particular importance for children exposed to current violent conflicts, with no end in sight.

Despite the improved quantity and quality of studies in global mental health [36, 49], this review of reviews still found evidence of widespread poor quality research especially at the basic services and security level of intervention. This highlights the ongoing friction between neat clinical research on manualised treatment interventions for PTSD and humanitarian agencies’ focus on delivering basic services and community support [20, 50]. Future research should target these wider public mental health and child protection interventions, to keep up with and reflect activities in the field. The global coverage of psychosocial intervention research for children also highlighted gaps, particularly alarmingly in countries with some of the most brutal armed conflicts in contemporary history. The Uppsala Conflict Data Program [51] identified that the highest number of fatalities due to armed conflict, non-state conflict and one-sided violence since 2015 has been in Syria, a country which was identified in this review as not having had a single intervention study conducted. This limits the generalisability of wider findings on interventions for children, and this is despite widespread humanitarian efforts, with an estimated 24 agencies implementing mental health and psychosocial support in the country [52]. This gap is likely to be due in part to the logistical, resource and security difficulties in conducting research in Syria, but has also been criticised as being a failure of the academic and humanitarian world to engage with each other to produce research that is of good quality but also responds to the short term and resource poor needs of the field [53].

### Strengths and limitations

This study is the first to systematically review the accumulating number of reviews of interventions for children affected by armed conflict, with an attempt to address theory-practice gaps by including grey and academic literature, and shifting the focus of the review to mechanisms and underlying processes as oppose to clinical effectiveness. The findings of this review are robust in that they draw on multiple studies, in diverse and widely geographically distributed countries and regions and large sample sizes of participating children, adolescents and young people, numbering well over 30,000. The review of reviews is limited, however, by a lack of description and statistical testing of mechanisms of change in primary research. Seven reviews were not included in the analysis as the mechanisms underlying included interventions were not addressed at all, and 5 of the mechanisms were supported by a poor quality of evidence, such as case studies or clinical experience and observation. Furthermore, 5 out of the 13 included reviews were of poor methodological quality and only 2 high quality reviews were found. Within these high quality reviews, only two primary studies were identified by the reviewers that statistically tested mechanisms of change [54, 55]. There is therefore not enough evidence to empirically support the majority of the mechanisms identified in this review, rather, the review highlights possible mechanisms, identified predominantly through program evaluations, that require scientific testing. In addition to this limitation, the wide scope of this review of reviews, which was intended to identify underlying mechanisms of change that transcend intervention techniques or project aims, does limit the possibilities of using the findings to inform targeted interventions for particular symptom clusters or mental disorders. The authors also note conceptual difficulty in differentiating mechanisms from therapeutic activities and protective factors. This is partly due to conceptual overlap in which subjective judgments had to be made, although it is hoped this subjectivity was reduced through a clear operational definition and duplicate analysis. This difficulty is also due to the lack of transparency in intervention research on their assumed mechanisms of change, with a number of reviews highlighting poor and vague descriptions of interventions in primary studies (e.g. [15]). Wider research on mechanisms of change in psychotherapy has highlighted this same limitation, with recommendations for future research including the assessment of multiple mechanisms in intervention research, establishing timelines for proposed mechanisms, and clear differentiation between moderators and mediators [28]. Finally, this review may have missed the identification of some reviews due to time and resource restrictions which led to limiting the number of databases searched and using an abstract and keyword search only.

### Implications

The findings of this review can help to inform caregivers and non-specialist practitioners in the humanitarian psychosocial field about the role of family and caregiver capacity building, family and caregiver relationship strengthening, and problem solving in non-specialist interventions, as well as therapeutic rapport during specialised interventions, in promoting positive change in the mental health, wellbeing, and resilience of children and adolescents affected by war. The findings of the review have also clearly identified the need for further research, with the majority of mechanisms assumed to work in the field supported by poor to moderate empirical evidence. Specifically, research that statistically tests proposed mechanisms of change, through mediational or component analysis, and with detailed timelines and robust measurements of mechanisms, is needed. Finally, this review has highlighted a need to understand the theoretical mechanism of trauma processing within the context of prolonged and ongoing exposure to war.

## Conclusions

In conclusion, it is hoped that the findings of this review of reviews can be of practical use in the frontline humanitarian field, particularly in regards to the continued use or implementation of the well-supported mechanisms identified in the review. Beyond this, the findings serves to highlight and encourage further research on non-specialist mechanisms of change for the wellbeing, resilience and mental health of children and adolescents affected by war. Future research that reflects the diversity of intervention aims and tests mechanisms statistically, is urgently required in order to be able to empirically support the role of the assumed mechanisms drawn on daily in the field.

## Additional files

Additional file 1: Figure S1. Flowchart showing the search process. (DOCX 58 kb)

Additional file 2: Table S1. Excluded studies based on abstract or full text. (DOCX 21 kb)

Additional file 3: Table S2. Supporting evidence and quality assessment for cited mechanisms of change. (DOCX 22 kb)

## Abbreviations

AMSTAR: A Measurement Tool to Assess Systematic Review
IASC: Inter-Agency Standing Committee
IFRC: International Federation of Red Cross and Red Crescent Societies
KidNET: Narrative Exposure Therapy for children
MSF: Médecins Sans Frontières
PILOTS: Published International Literature On Traumatic Stress
PRISMA: Preferred Reporting Items for Systematic Reviews and Meta-Analyses
PTSD: Posttraumatic Stress Disorder
TF-CBT: Trauma-Focused Cognitive Behavioural Therapy
