# Supplementary material for: Mechanisms of change for interventions aimed at improving the wellbeing, mental health and resilience of children and adolescents affected by war and armed conflict: a systematic review of reviews
Source: Confl Health. 2018 May 9;12:15. doi: 10.1186/s13031-018-0153-1 (PMC5941634; doi:10.1186/s13031-018-0153-1)
Supplement: Supplementary file 1 — Figure S1. Flowchart showing the search process. (DOCX 58 kb) [file 13031_2018_153_MOESM1_ESM.docx]

Additional file 1

Figure S1: Flowchart showing the search process

193 records identified in Cochrane library search

214 records identified in grey literature search

519 records identified in PILOTS

1,433 records identified in PubMed search

20 records selected by title

12 records selected by title

3 records selected by title

16 records selected by title

4 records included

3 records selected by abstract

1 records selected by abstract

4 records selected by abstract

0 records included

1 record included

4 records included

6 selected by reference list of included study search, 3 included

13 included in total
