# Supplementary material for: Mechanisms of change for interventions aimed at improving the wellbeing, mental health and resilience of children and adolescents affected by war and armed conflict: a systematic review of reviews
Source: Confl Health. 2018 May 9;12:15. doi: 10.1186/s13031-018-0153-1 (PMC5941634; doi:10.1186/s13031-018-0153-1)
Supplement: Supplementary file 2 — Table S1. Excluded studies based on abstract or full text. (DOCX 21 kb) [file 13031_2018_153_MOESM2_ESM.docx]

Additional file 2

Table S1: Excluded studies based on abstract or full text

| **Publication details** | **Source** | **Excluded by abstract or full text** | **Main reason for exclusion** |
| --- | --- | --- | --- |
| Pynoos RS, Nader K. Prevention of psychiatric morbidity in children after disaster. Stress & Health. 1989;5(1):233-279. doi: 10.1002/smi.2460050107. | PILOTS | Full text | No war –affected population sub-group analysis |
| Stallard P, Salter E . Psychological Debriefing with Children and Young People Following Traumatic Events. Clinical Child Psychology and Psychiatry. 2003;8(4): 445-457. doi: 10.1177/13591045030084003. | PILOTS | Full text | No war –affected population sub-group analysis |
| LaGreca AM, Silverman KS. Treatment and prevention of posttraumatic stress reactions in children and adolescents exposed to disasters and terrorism: what is the evidence? Child Development Perspectives. 2009;3:4-10. doi: 10.1111/j.1750-8606.2008.00069.x. | PILOTS | Full text | No war –affected population included |
| Patel N, Kellezi B, Williams ACDC. Psychological, social and welfare interventions for psychological health and well-being of torture survivors (Review). Cochrane Database Syst Rev. 2014;11. doi: 10.1002/14651858.CD009317.pub2. | Cochrane Library for Systematic Reviews | Abstract | No children or adolescents included |
| Coren E, Hossai R, Pardo J, Bakker B. Interventions for promoting reintegration and reducing harmful behaviour and lifestyles in street-connected children and young people (Review). Cochrane Database Syst Rev. 2016;1. doi: 10.1002/14651858.CD009823.pub3. | Cochrane Library for Systematic Reviews | Abstract | No war –affected population sub-group analysis |
| Bakker D, Kazantzis N, Rickwood D, Rickard N. Mental health smartphone apps:  Review and evidence-based recommendations for future developments. JMIR Ment Health. V;3(1). doi: 10.2196/mental.4984. | PubMed | Abstract | No war –affected population sub-group analysis |
| Pfefferbaum B, North CS. Child disaster mental health services: A review of the system of care, assessment approaches, and evidence base for intervention.  Curr Psychiatry Rep. 2016;18(5). doi: 10.1007/s11920-015-0647-0. | PubMed | Abstract | No war –affected population sub-group analysis |
| Dray J, Bowman J, Wolfenden L, Campbell E, Freund M, Hodder R, Wiggers J. Systematic review of universal resilience interventions targeting child and adolescent mental health in the school setting: review protocol. Syst Rev. 2015;29. doi: 10.1186/s13643-015-0172-6. | PubMed | Abstract | No review (protocol only) |
| Pryjmachuk S, Elvey R, Kirk S, Kendal S, Bower P, Catchpole R. Developing a model of mental health self-care support for children and young people through an integrated evaluation of available types of provision involving systematic review, meta-analysis and case study. Health Services and Delivery Research. 2014;2(18). | PubMed | Abstract | No war –affected population sub-group analysis |
| Keeshin BR, Strawn JR. Psychological and pharmacologic treatment of youth with posttraumatic stress disorder: An evidence-based review. Child Adolesc Psychiatr Clin N Am. 2014;23:399-411. doi: 10.1016/j.chc.2013.12.002. | PubMed | Abstract | No war –affected population sub-group analysis |
| Barry MM, Clarke AM, Jenkins R, Patel V. A systematic review of the  effectiveness of mental health promotion interventions for young people in low  and middle income countries. BMC Public Health. 2013;13. doi: 10.1186/1471-2458-13-835. | PubMed | Abstract | No war –affected population sub-group analysis |
| Tol WA, Song S, Jordans MJ, Annual Klasen H, Crombag AC. What works where? A systematic review of child and adolescent mental health interventions for low and middle income countries. Soc Psychiatry Psychiatr Epidemiol. 2013;48:595-611. doi: 10.1007/s00127-012-0566-x. | PubMed | Abstract | No war –affected population sub-group analysis |
| Dimitry L. A systematic review on the mental health of children and adolescents in areas of armed conflict in the Middle East. Child Care Health Dev. 2012;38:153-61. doi: 10.1111/j.1365-2214.2011.01246.x. | PubMed | Abstract | No review of interventions or mechanisms (prevalence only) |
| Tol WA, Kohrt BA, Jordans MJ, Thapa SB, Pettigrew J, Upadhaya N, de Jong JT. Political violence and mental health: a multi-disciplinary review of the literature on Nepal. Soc Sci Med. 2010;70:35-44. doi: 10.1016/j.socscimed.2009.09.037. | PubMed | Abstract | No war –affected population sub-group analysis |
| Walsh DS. Interventions to reduce psychosocial disturbance following humanitarian relief efforts involving natural disasters: an integrative review. Int J Nurs Pract. 2009;15:231-40. doi: 10.1111/j.1440-172X.2009.01766.x. | PubMed | Abstract | No child or adolescent sub-group analysis |
| Amstadter AB, Broman-Fulks J, Zinzow H, Ruggiero KJ, Cercone J. Internet-based interventions for traumatic stress-related mental health problems: A review and suggestion for future research. Clin Psychol Rev. 2009;29:410-20. doi: 10.1016/j.cpr.2009.04.001. | PubMed | Abstract | No war –affected population sub-group analysis |
| Wiley-Exley E. Evaluations of community mental health care in low- and  middle-income countries: a 10-year review of the literature. Soc Sci Med. 2007;64:1231-41. doi: 10.1016/j.socscimed.2006.11.009. | PubMed | Abstract | No war –affected population sub-group analysis |
| De Berry J. Community Psychosocial Support in Afghanistan. Intervention. 2004;2:143 –151. | War Trauma Foundation | Full text | No review (intervention description only) |
| Omowumi Babatunde A. Harnessing traditional practices for use in the reintegration of child soldiers in Africa: examples from Liberia and Burundi. Intervention. 2014;12:379–392 | War Trauma Foundation | Full text | No review (intervention description only) |
| Williamson J. The disarmament, demobilization and reintegration of child soldiers: Social and psychological transformation in Sierra Leone. Intervention. 2006;4:185–205. | War Trauma Foundation | Full text | No review (intervention description only) |
| Ostadestraat V. War Child Holland: Evidence from the Field. Amsterdam : War Child (Holland); 2013. | War Child (Holland) | Full text | No review (intervention evaluation only) |
| MSF. Psychosocial and mental health interventions in areas of mass violence: A community-based approach. Amsterdam: MSF; 2011. | MSF | Full text | No review (intervention description only) |
| MSF. Rwanda: Providing psychological and medical care. MSF International; 2017. | MSF | Full text | No children or review |
| MSF. Healing invisible wounds in Nigeria: MSF uses art and counseling to treat children's trauma. MSF International, 2017. | MSF | Full text | No review (intervention evaluation only) |
| MSF. Palestinian Territories: Providing crucial mental health and medical care. MSF International, 2017. | MSF | Full text | No review (intervention evaluation only) |
| Wangara R, Ogwang M. Djibouti Population Movement DREF Review. IFRC; 2016. | IFRC | Full text | No psychological interventions or mechanisms |
| Faller M. TC Pam-Livelihood Peer Review Vanuatu. IFRC; 2016. | IFRC | Full text | No psychological interventions or mechanisms |
| IFRC. Children affected by armed conflict: The Children’s Resilience Programme. Copenhagen: Danish Red Cross; NR. | IFRC PS Centre | Full text | No review of interventions or mechanisms (handbook) |
| IFRC. Psychosocial Support for Youth  in Post-Conflict Situations. Copenhagen: Danish Red Cross; 2014. | IFRC PS Centre | Full text | No review of interventions or mechanisms (handbook) |
| IFRC. Strengthening resilience: A global selection of psychosocial interventions. Copenhagen: Danish Red Cross; 2014. | IFRC PS Centre | Full text | No sub-group of children or adolescents affected by conflict |
| IFRC. The Resilience Programme  for Young Men. Copenhagen: Danish Red Cross; 2015. | IFRC PS Centre | Full text | No review of interventions or mechanisms (handbook) |
| IFRC. Coping with Crisis. Copenhagen: Danish Red Cross; 2015. | IFRC PS Centre | Full text | No review (intervention description) |
| Save the Children. Invisible Wounds: The impact of six years of war on the mental health of Syria’s children. London: Save the Children; 2017. | Save the Children | Full text | No review of interventions or mechanisms |
| Save the Children. How to Help Children Cope with Transition in Disasters. Connecticut: Save the Children; NR. | Save the Children | Full text | No war –affected population |
| Save the Children. A devastating toll: The impact of three years of war on the health of Syria’s children. London: Save the Children; 2014. | Save the Children | Full text | No psychological interventions or mechanisms |

Acronyms: IFRC International Federation of Red Cross and Red Crescent Societies; MSF Médecins Sans Frontières; NR Not Reported; PS Centre Psychosocial Centre
