# Supplementary material for: Mechanisms of change for interventions aimed at improving the wellbeing, mental health and resilience of children and adolescents affected by war and armed conflict: a systematic review of reviews
Source: Confl Health. 2018 May 9;12:15. doi: 10.1186/s13031-018-0153-1 (PMC5941634; doi:10.1186/s13031-018-0153-1)
Supplement: Supplementary file 3 — Table S2. Supporting evidence and quality assessment for cited mechanisms of change. (DOCX 22 kb) [file 13031_2018_153_MOESM3_ESM.docx]

Additional file 3

Table S2: Supporting evidence and quality assessment for cited mechanisms of change

| **Mechanisms** | | **Cited reviews** | **Supporting evidence** | **Quality rating** |
| --- | --- | --- | --- | --- |
| **BASIC SERVICES AND SECURITY** | | | | |
| 1 | Creating safety and protection from harm | Ager et al. [17] | - Improvements in protection outcomes post intervention reported by:   - One end of program evaluation study   - One outcome study   - Two service evaluations   - One internal evaluation report   - One assessment report - Improvements in social and emotional wellbeing post intervention reported by:   - One evaluation report | Low: Program evaluations |
| 2 | Playing | Apfel and Simon [1]  Betancourt et al. [14] | - Improved resilience reported by two case studies and one intervention manual - Improvements in child wellbeing reported by one cross-sectional study - Improved self-confidence and emotional regulation post intervention reported from one qualitative study | Low: Case or cross-sectional studies |
| **STRENGTHENING FAMILY AND COMMUNITY SUPPORT** | | | | |
| 3 | Community capacity building | Ager et al. [17]  Apfel and Simon [1]  Peltonen & Punamäki [6] | - Increased knowledge of protection systems post intervention reported by:   - Two end of program evaluation studies   - One internal evaluation report   - One service evaluation   Poor evidence for increased reporting of abuse post intervention   - Re-establishing a sense of order and sanity, supported by the authors own clinical experience - Reduction in PTSD, and improved psychosocial wellbeing, reported by 5 pre/post evaluations | Low: Program evaluations |
| 4 | Increasing social support | Apfel and Simon [1]  Peltonen & Punamäki [6] | - Improved resilience reported by one case study - Reduction in PTSD, and improved psychosocial wellbeing, reported by 7 pre/post evaluations | Low: Program evaluation and clinical experience |
| 5 | Family and caregiver capacity building | Apfel and Simon [1]  Jordans, Pigott and Tol [36] | - Improved ability of caregivers to provide consistent and reliable care supported by the authors own clinical experience - Reduction in depression, PTSD, and anxiety symptoms, and improved hope, reported by two multiple n=1 studies | High: Statistical testing of mechanism |
| 6 | Family and caregiver relationship strengthening | Apfel and Simon [1]  Betancourt et al. [14]  Jordans, Pigott and Tol [36] | - A buffering affect against further traumatic experience reported by three long-term retrospective studies, one prospective study, and one follow-up study - Modest positive effects on maternal mental health, and children's psychosocial functioning and mental health post intervention reported by one controlled trial - Reduction in depression, PTSD, and anxiety symptoms, and improved hope, reported by two multiple n=1 studies | High: Statistical testing of mechanism |
| 7 | Engaging with values, traditions, religious and non-religious beliefs, and ideologies | Apfel and Simon [1]  Betancourt et al. [14]  Tol, Song and Jordans [11] | - Rebuilding morale and healing, supported by one cross-sectional study and the authors own clinical experience - Improved ability to maintain the right to be alive despite suicidal despair, reported by one case study and a cross-sectional study - Improved drive to survive, supported by authors own clinical experience - Preserved community and personal restitution, reported by two memoirs, once by a journalist and one by a clinician - Enabled empowerment, reported by one program evaluation and anecdotal evidence - Improved reintegration of child soldiers into communities post ritual reported in one observational study, but no improvements for mental health were reported - Improved wellbeing reported by 15 qualitative studies | Low: Qualitative studies or clinical experience |
| **FOCUSED NON-SPECIALIST SUPPORT** | | | | |
| 8 | Learning about the presenting problem, medication, and how to access services (psychoeducation) | Betancourt et al. [14] | - Improved medication compliance and access to services post intervention reported by one RCT - Reduced distress post intervention reported by one RCT, when combined with skills building and supportive counsellor contact | Moderate: Statistic testing but of intervention not mechanism |
| 9 | Learning stress management skills | Peltonen & Punamäki [6] | - Reduction in PTSD, and improved psychosocial wellbeing, reported by 7 pre/post evaluations | Moderate: Statistic testing but of intervention not mechanism |
| 10 | Emotional regulation and bearing negative emotions | Apfel and Simon [1]  Peltonen & Punamäki [6] | - Improved chances of survival and resilience supported by authors own clinical experience - Reduction in PTSD, and improved psychosocial wellbeing, reported by 12 pre/post evaluations | Low: Program evaluation and clinical experience |
| 11 | Problem solving | Jordans, Pigott and Tol. [36] | - Reduction in depression, PTSD, and anxiety symptoms, and improved hope, reported by two multiple n=1 studies | High: Statistical testing of mechanism |
| 12 | Learned helpfulness | Apfel and Simon [1]  Betancourt et al. [14] | - Reduced sense of helplessness, supported by one cross-sectional study, the authors own clinical experience, and three qualitative program evaluations - Correlational evidence of a role of attachment to the object of care (a doll) on wellbeing from one RCT | Moderate: Statistical testing but of intervention not mechanism |
| **SPECIALIST SUPPORT** | | | | |
| 13 | Adverse mechanism: Targeted interventions | Apfel and Simon [1] | - Risks alienating participants, supported by authors own clinical experience | Low: Clinical experience |
| 14 | Trauma processing through narratives, exposure, dreaming or play | Apfel and Simon [1]  Betancourt et al. [14]  Jordans, Pigott and Tol. [36]  Peltonen & Punamäki [6] | - Helps to re-organize, recollect, narrate and integrate memories, reported by one case study and retrospective interviews by journalists - Reduction in PTSD symptoms post intervention reported by 4 RCTs - Reduction in depression, PTSD, and anxiety symptoms, and improved hope, reported by two multiple n=1 studies - Reduction in PTSD, and improved psychosocial wellbeing, reported by 4 pre/post evaluations | Moderate: Statistical testing but of intervention not mechanism |
| 15 | Restructuring unhelpful cognitions and appraisals | Peltonen & Punamäki [6] | • Reduction in PTSD, and improved psychosocial wellbeing, reported by 16 pre/post evaluations | Moderate: Statistical testing but of intervention not mechanism |
| 16 | Therapeutic rapport | Tol, Song & Jordans [11] | - Reduction in depression, PTSD, and anxiety symptoms, and improved hope, reported by two multiple n=1 studies - Adverse mechanism of moralistic counselling: Negative impact on depression, PTSD, and anxiety symptoms, and hope, reported by two multiple n=1 studies | High: Statistical testing of mechanism |
